# Vaginal treatment with lactic acid gel delays relapses in recurrent urinary tract infections: results from an open, multicentre observational study

**DOI:** 10.1007/s00404-021-06040-8

**Published:** 2021-03-27

**Authors:** Ruth Diebold, Bettina Schopf, Holger Stammer, Werner Mendling

**Affiliations:** 1 Dr. Kade Pharmazeutische Fabrik GmbH, Berlin, Germany; 2 Pharmalog Institut für klinische Forschung GmbH, Ismaning, Germany; 3 German Centre for Infections in Obstetrics and Gynecology, Wuppertal, Germany

**Keywords:** Urinary tract infection, Uropathogen, Lactic acid gel, Preventive treatment, Relapse reduction

## Abstract

**Purpose:**

The main objective of this open, prospective, multicentre, observational study is to investigate the relapse rate and tolerability of lactic acid gels in adult female patients with recurrent urinary tract infections during routine practice.

**Methods:**

Data were collected from patients undergoing intermittent short courses of intravaginal treatment with lactic acid gel for prevention of recurrent urinary tract infections. The observation period for individual patients was 4 months, aimed at covering four short courses of intravaginal treatment. Data on UTI relapses, tolerability, handling and satisfaction with the treatment were collected via patient diaries and physician assessments and comprised any adverse events (AEs).

**Results:**

In total, 72 patients were treated. During the last 12 months prior to the study, patients had on average 4.0 UTIs. In the 4 months after commencing treatment, 63.5% of patients had no recurrence of UTI symptoms. Overall efficacy was rated by physicians as ‘excellent/good’ for 96.7% of patients. The patients’ overall acceptance of local treatment was high with 94.1% being ‘(very) satisfied’. Similarly, handling was rated as ‘(very) easy’ by 94.2% of patients. The tolerability was assessed as ‘highly tolerable/tolerable’ by over 98% of patients and physicians alike. Safety analyses reported six AEs of mild intensity, all of which had resolved by the end of the study.

**Conclusion:**

Treatment with lactic acid gel may increase resilience against uropathogens, possibly preventing the need for antibiotic prevention of recurrent urinary tract infections. Treatment was positively assessed by the patients. The physician assessments corroborate these findings.

**Trial registration number and date of registration:**

DRKS00016760, 18.02.2019.

## Introduction

Urinary tract infections (UTIs) are presumably one of the most common bacterial infections [1]. Even though the prevalence is difficult to assess due to the self-limiting nature of UTIs and self-treatment, UTIs are possibly the most common urological disorder [2, 3], which affects women disproportionately [1, 4]. UTIs are clinically distinguished between uncomplicated and complicated cases [3]. While in uncomplicated UTIs neither structural or neurological urinary tract abnormalities, nor relevant renal disorders occur [2], complicated infections often manifest as cystitis or pyelonephritis.

By far the most common pathogen is *Escherichia coli*, responsible for approximately 85% of UTIs, with other pathogens such as *Enterococcus faecalis*, *Staphylococcus saprophyticus*, *Klebsiella pneumoniae*, *Proteus mirabilis* occurring considerably less frequently [5]. Recurrent UTIs (rUTIs), defined as at least three UTIs within 12 months or at least two UTIs within 6 months [6], are a great burden for the patients and have an evident, negative impact on the patients’ quality of life [7–9].

The healthy vaginal microbiota is dominated by *Lactobacillus* species, which produce bacteriocins, surfactants, and other antimicrobial products and generally lower the vaginal pH by producing lactic acid [10]. During a UTI, *Lactobacilli* are depleted by colonization with (usually intestinal) *E. coli* at the vaginal introitus and periurethra. The subsequent increase of pH levels facilitates proliferation of *E. coli* and creates beneficial conditions for *E. coli*, while being more detrimental to the naturally occurring *Lactobacilli*, respectively [11–13].

Some treatments for rUTIs rely on probiotics, which re-inoculate the microbiota with *Lactobacilli* to shift conditions back in favour of those lactic acid producing bacteria [14, 15]. An alternative method is the vaginal application of lactic acid instead of *Lactobacilli* [16]. This fosters proliferation of the natural occurring, already present *lactobacilli* by lowering the pH level and thus restoring the natural, acidic environment.

In this observational study, lactic acid was administered as a gel in a prefilled, single-use applicator for intravaginal use [17]. Treatment occurred in short courses of 2 to 3 treatment days after each menstruation in premenopausal women and with monthly intervals in postmenopausal women. The aim of the study was to obtain real-world data on relapse prevention with a lactic acid gel in women suffering from rUTIs. Key aspects of the study were the percentage of relapses of UTIs and the tolerability of lactic acid gel as prophylaxis in adult females with uncomplicated rUTIs in daily practice. Here, we report on the results after four short courses of intravaginal treatment over 4 months of treatment.

## Materials and methods

### Study design

PANAMA is a multicentre, prospective, uncontrolled, single group, observational study for the prevention of relapses in rUTI with lactic acid gel (KadeFungin® Milchsäurekur). The study was performed in Germany and aimed to enrol approximately 60–80 female patients from about 30 doctor’s offices (study centres). Recruitment was anticipated to last 5 months and was planned to begin in February 2019. About two to three patients per centre were desired. Primary care physicians (general practitioners) and secondary care physicians (specialists for gynaecology and urology), who are familiar with the symptoms and treatment of acute episodes of uncomplicated rUTI, were invited to participate in the study.

The study was carried out in accordance with the ethical principles laid down in the current revision of the World Medical Association's Declaration of Helsinki as amended by the 64th General Assembly in Fortaleza in 2013 [18] and international standards for good clinical practice in medical device studies defined in ISO 14155:2011 [19]. All study-relevant documents were submitted to the Ethics Committee (EC) responsible for the applicant /participating physician, in accordance with article 15(1) of the (Model) Professional Code for Physicians in Germany (MBO-Ä) [20] to obtain a consultation for professional rights and ethics of physicians in this study according to § 23b of German Medical Device Law (MPG).

Women could be included in the study if they were ≥ 18 years of age and had a medically confirmed history of recurrent acute uncomplicated urinary tract infection (i.e., ≥ 2 UTIs within the last 6 months or ≥ 3 UTIs within the last 12 months). A list of common risk factors associated with complicated urinary tract infection was included in the observation plan and the occurrence of these was queried. Women with asymptomatic bacteriuria were not included in the observational study. Patients were excluded from the study if they had a known hypersensitivity to any of the ingredients of KadeFungin® Milchsäurekur. Although the aim of the study was to document the effect on prevention of rUTI, urinary tract infection at inclusion was not an exclusion criterion. Informed consent was obtained from all patients in the eCRF.

KadeFungin® Milchsäurekur is a non-prescription CE-certified medical device class IIa filled with lactic acid gel for intravaginal application. With a pH value of four it resembles the optimal natural physiological condition. Thereof it is intended for regeneration and stabilization of the vaginal microbiota. With each applicator, 2.5 ml lactic acid (corresponding to 150 mg of S-lactic acid) is released deep into the vagina. Recommendation is to use one applicator filling once daily before bedtime for 2 to 3 days after each menstruation/in monthly intervals.

Demographic and baseline characteristics were recorded: age (years), year of birth, confirmation of female gender and inclusion diagnosis, number of UTIs during the 12-month period prior to visit D1 (including current episode of UTI, if applicable), previous treatment of rUTIs, other current medical conditions/treatment (antibiotics or other) related to UTI), dispense of diary.

Patients were asked to keep a monthly diary for 4 months after D1 on the following aspects: occurrence of UTI in the previous month (medical confirmation of UTI was not explicitly required), onset/end of UTI (if occurred), how the UTI was treated (antibiotics or others) and use of KadeFungin® Milchsäurekur according to instructions for use (yes/no). After the 4-month treatment period, they rated once the treatment satisfaction (rating options: very satisfied, satisfied, moderately satisfied, dissatisfied), the product handling (rating options: very easy, easy, moderate, difficult), the additional moisturisation (rating options: very pleasant, pleasant, neutral, unpleasant, very unpleasant) and the local tolerability of KadeFungin® Milchsäurekur (rating options: highly tolerable’, ‘tolerable’, ‘moderately tolerable’, ‘intolerable’). In addition, all adverse events were recorded. If there was no face-to-face visit, the patient could be asked to return the diary to the physician by post, or the physician could conduct a phone call and request the information in the diary, otherwise the patient was counted as lost to follow-up.

An optional visit D2 (approximately 4 months, but no later than 4.5 months after D1) could be performed to assess the patient’s health status, including a vulvovaginal examination with pH measurement (optional), as well as assessment of local tolerability (rating options: ‘highly tolerable’, ‘tolerable’, ‘moderately tolerable’, ‘intolerable’) and presence or absence of erythema, oedema, vulvovaginal itching, others (specify).

After contact with the patient, the physician was asked to indicate whether an assessment of tolerability/efficacy was possible. The assessment of tolerability/efficacy ranged from excellent, good, moderate, bad and very bad.

In accordance with the design of the study, the measurement of vaginal pH at D1 and D2 was left to the decision of the treating physician.

The primary endpoint of the study was the % of patients without UTI-relapse within 4 months after visit D1 based on diary data. Secondary endpoints with regard to efficacy were the % of patients without relapse within 1, 2 and 3 months after visit D1, time to first relapse, number of relapses within 4 months after D1, mean change of vaginal pH value from baseline to D2, physicians’ global judgments of efficacy, patients’ assessments of treatment satisfaction, patients’ assessments of additional moisturizing effect, patients’ adherence to the mode of application described in the user manual, and patients’ assessments of product handling. Secondary endpoints with regard to safety and tolerability were the type, frequency and severity of adverse events (AEs), adverse device effects (ADEs) or device deficiencies [21], patients’ judgments of local tolerability and physicians’ global judgements of tolerability.

### Statistics

Population required to demonstrate a relevant effect in relation to the primary endpoint was calculated using the PASS 11 sample size software [22]. In case of clinically relevant patient benefit from the application of the gel, a sample size of 60–80 patients is sufficient to estimate these effects with adequate statistical precision, i.e., ± 10% in the 90% confidence interval range for the primary end point. Depending on the scope of the analysis, different patient datasets were used. The safety analysis set (SES) included all patients who administered the medical device under investigation and for whom any post-baseline safety data were documented in the diary and/or eCRF. The full analysis set (FAS) included all patients of the SES who documented any post-baseline information on the reoccurrence of UTI in the diary. The complete case (CC) cohort included all patients of the FAS who documented four treatment courses in the diary (one course per month) and had an individual observation time of ≥ 106 days (= 4 months–2 weeks tolerance as period between date of visit D1 and date of patient’s last entry in the diary). Primary and secondary endpoints were analysed using the FAS and CC cohort. Safety endpoints were evaluated using the SES. If applicable, 95 and 90% Wilson confidence intervals were calculated for primary endpoint analysis. Comparison of subgroup categories was performed using the Fisher’s exact test for 2 × 2 categorical data. In the following description of results, *N* describes the total number of patients, *N*_valid_ represents the number of patients with valid observations and *n* the respective observed frequencies counted for the described event.

## Results

**Fig. 1:**
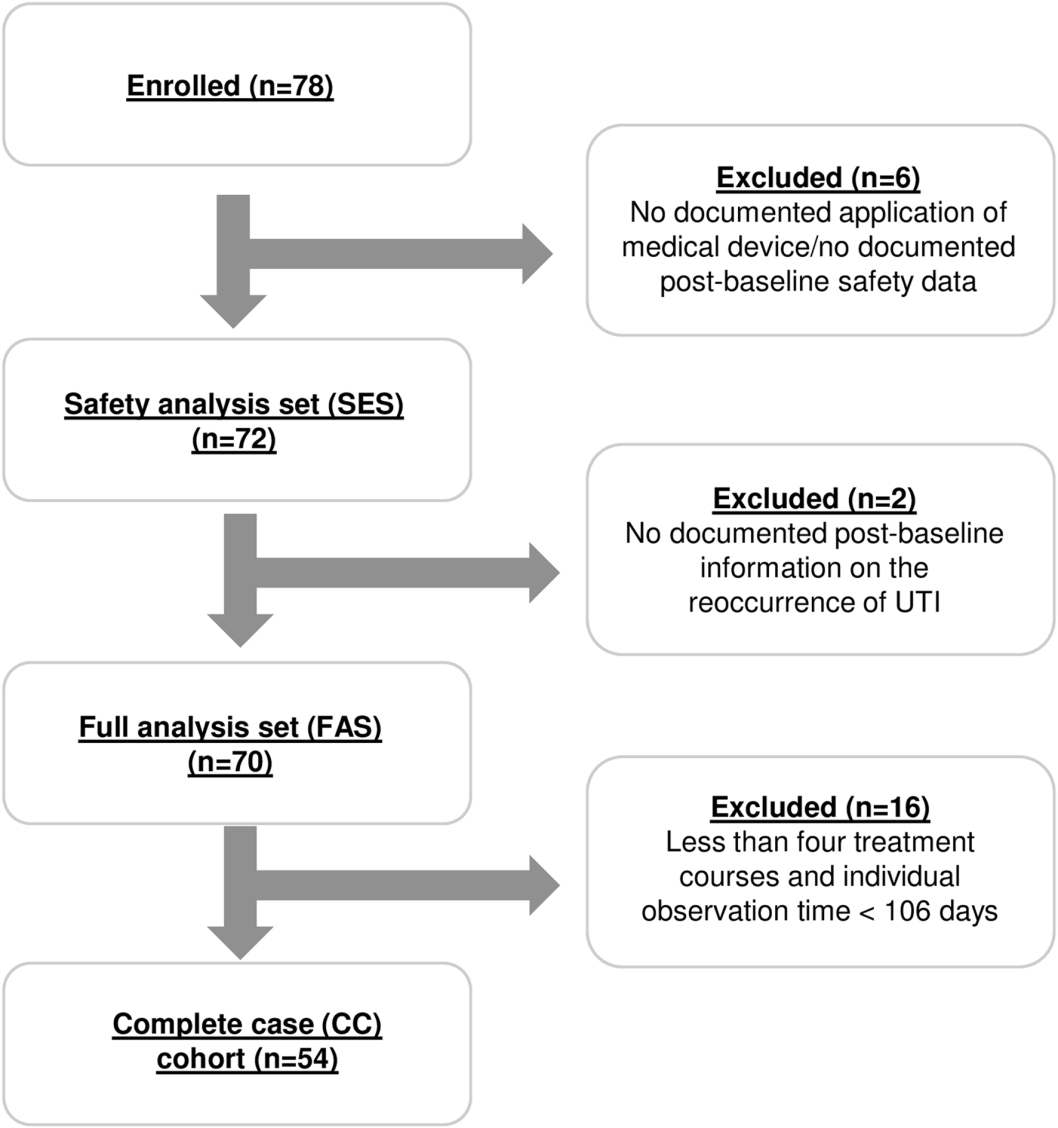
Patient flow chart and analysis sets

In total, 78 women with rUTI were enrolled in the study. For reasons unrelated to treatment, six patients were excluded from the safety analyses set (SES: *N* = 72, 100%), as well as two from the full analysis set (FAS: *N* = 70, 100%). Another 16 patients were excluded from the CC cohort. (CC cohort: *N* = 54, 100%) (Fig. 1).

Two third of patients were less than 50 years old (66.7%). In the SES the % with 2–3 UTIs was 48.6, 4–5 UTIs occurred in 34.7% and six or more in 16.7%, with a mean number of 4.0 UTIs (SD 1.9; range 2–10 UTIs). Risk factors associated with a complicated UTI were not reported for any of the 72 patients at baseline.

Fifty one patients (70.8%) had been treated with antibiotics (48 patients, 66.7%) or herbal medicine (3 patients 4.2%) during the 3-month period prior to visit D1. Concomitant medication for acute UTI at inclusion was used in eight (11.1%) (Table 1). During the 4-month observation period, patients did not document any concomitant medication.

**Table 1:** Concomitant medication for acute UTI at inclusion reported at visit D1 (SES)

	Number (%) of patients
Total patients in the SES	72 (100%)
Total patients with concomitant medication for acute UTI at inclusion	8 (11.1)
Medication as reported in the eCRF	
Ciprobay 500	1 (1.4)
Ciprofloxacin 250	1 (1.4)
Antibiotics (not specified)	4 (5.6)
Nitrofurantoin	1 (1.4)
Fluomizin	1 (1.4)

The median study duration was 126.0 days (range 79–179 days) for both the SES (*N*_valid_ = 71) and the FAS (*N* = 70; 100.0%). In the CC cohort, the median study duration was 126.0 days (range 119–145 days). The maximum study duration of 179 days was recorded for one patient. The mean number of treatment courses within 4 months was 3.6 (SD 1.0) in the SES, 3.7 (SD 0.8) in the FAS and 4.0 (SD 0.0) in the CC cohort. The % of treatment-compliant patients was over 90%. Sixty three patients (94.0%) were treatment-compliant in the FAS (94.0% of 67 patients with non-missing data) and all 54 patients (100%) in the CC cohort (treatment-compliant patients used KadeFungin® Milchsäurekur once per month over 4 months).

**Fig. 2:**
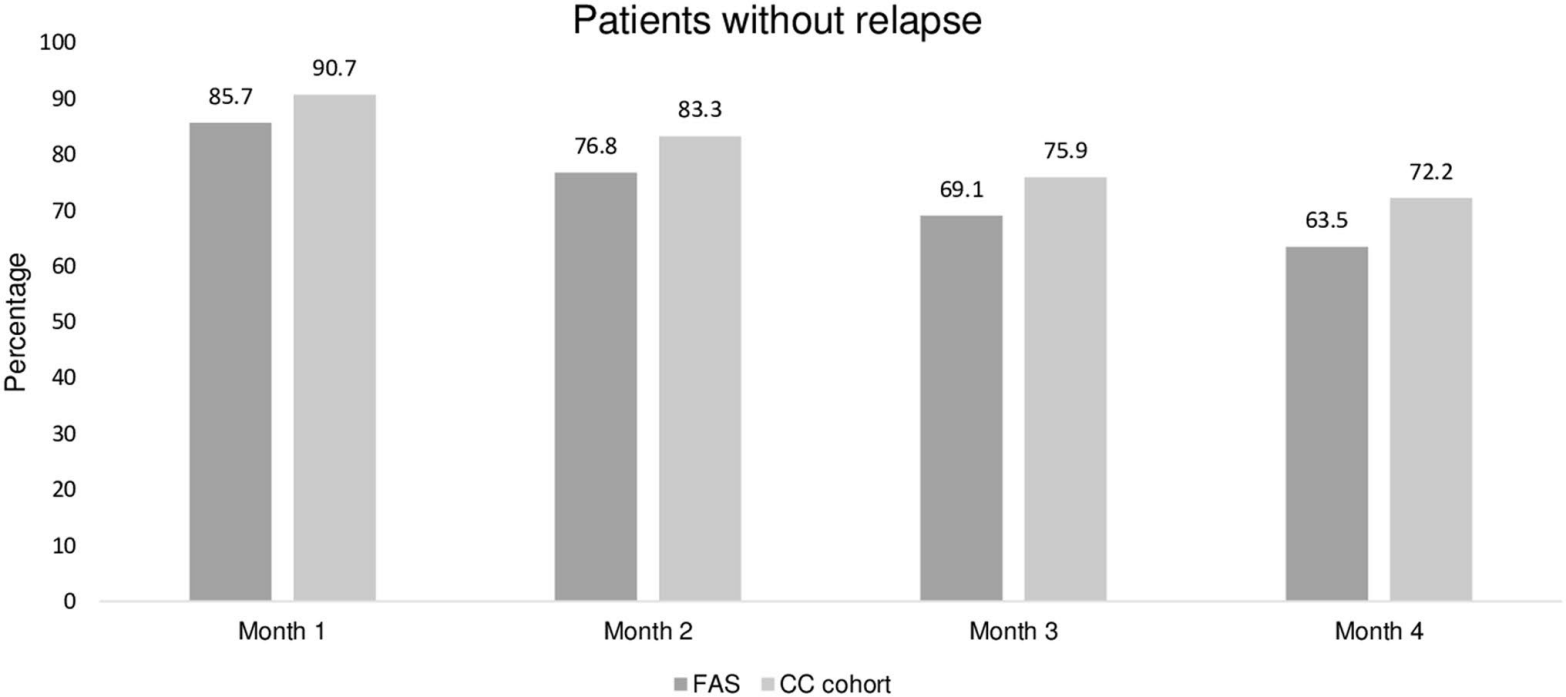
Percentage of patients with no relapse of UTI during 1, 2, 3 and 4 months of observation in FAS (*N*_valid_ = 70/69/68/63, for periods of 1, 2, 3 and 4 months) and CC cohort (*N* = 54, 100% for all periods)

In the FAS (*N*_valid_ = 63), 63.5% of patients had no relapse of UTI symptoms within 4 months (95% Wilson CI between 51.2 and 74.3%). In the CC cohort (*N* = 54), it was 72.2% of patients (95% Wilson CI between 59.1 and 82.4%). The proportion of relapse-free patients in the FAS decreased from 85.7% after the first month, to 76.8 and 69.1% after the second and third month, respectively (FAS, *N*_valid_ = 70/69/68, 100%). The corresponding values in the CC cohort were 90.7, 83.3 and 75.9% (CC, *N* = 54, 100%) (Table 2, Fig. 2).

**Table 2:** Proportion of patients with and without relapse and 90/95% Wilson confidence intervals (FAS, CC cohort) within 4 months after D1

	Patients *N *(%)	Binominal proportion	95% Wilson confidence interval	
			Lower limit	Upper limit
Without relapse within 4 months after D1 (primary endpoint)				
FAS (*N*_valid_ = 63)				
Yes	40 **(63.5)**	0.635	0.5115	0.7428
No	23 (36.5)			
CC cohort (*N* = 54, 100%)				
Yes	39 **(72.2)**	0.722	0.5911	0.8238
No	15 (27.8)			
Without relapse within 3 months after D1 (secondary endpoint)				
FAS (*N*_valid_ = 68)				
Yes	47 **(69.1)**	0.691	0.5736	0.7883
No	21 (30.9)			
CC cohort (*N* = 54, 100%)				
Yes	41 **(75.9)**	0.759	0.6305	0.8536
No	13 (24.1)			
Without relapse within 2 months after D1 (secondary endpoint)				
FAS (*N*_valid_ = 69)				
Yes	53 **(76.8)**	0.768	0.6560	0.8519
No	16 (23.2)			
CC cohort (*N* = 54, 100%)				
Yes	45 **(83.3)**	0.833	0.7126	0.9098
No	9 (16.7)			
Without relapse within 1 month after D1 (secondary endpoint)				
FAS (*N* = 70, 100%)				
Yes	60 **(85.7)**	0.857	0.7566	0.9205
No	10 (14.3)			
CC cohort (*N* = 54, 100%)				
Yes	49 **(90.7)**	0.907	0.8009	0.9598
No	5 (9.3)

For patients that had at least one relapse of UTI symptoms in the 4-month observation period (FAS, 36.5%), the mean time to first relapse was 46.8 days, which had a high variance (median 41 days, range 8–116 days).

Subgroup analyses based on baseline characteristics showed no evidence that the % of patients being relapse-free after 4 month(s) differed by patient age (categories 18–49; ≥ 50 years) or the number of UTIs in the previous 12 months (categories 2–3; 4–5; > 6 UTIs). The two-sided *p* values of the Fisher’s exact test calculated for the FAS were all > 0.05 (Table 3).

**Table 3:** Subgroup analyses by baseline characteristics (previous UTIs and age)

Subgroups by category	Total patients per category	Patients without relapse *N* (%)	Binominal proportion	90/95% Wilson confidence interval		Fisher's exact test
				Lower limit	Upper limit	
Number of UTIs during the past 12 months						
2–3	34	22 (64.7)	0.647	0.5060 0.4791	0.7664 0.7851	^a^0.7691 ^b^0.1975 ^c^0.2348
4–5	21	15 (71.4)	0.714	0.5353 0.5004	0.8444 0.8619	
≥ 6	8	3 (37.5)	0.625	0.1612 0.1368	0.6520 0.6943	
Age [age]						
18–49	41	30 (73.2)	0.732	0.6062 0.5807	0.8285 0.8431	^d^0.0532
≥ 50	22	10 (45.5)	0.455	0.2947 0.2692	0.6244 0.6534	

Number (%) of patients without relapse within 4 months after D1 and *p* values for the Fisher’s exact test (FAS: *N*_valid_ = 63)

^a^*p* value for the comparison of UTI categories ‘2–3’ versus ‘4–5’

^b^*p* value for the comparison of UTI categories ‘4–5’ versus ‘ ≥ 6’

^c^*p* value for the comparison of UTI categories ‘2–3’ versus ‘ ≥ 6’

^d^*p* value for the comparison of age categories ‘18–49’ versus ‘ ≥ 50’

The same applied to the CC cohort (data not shown). When interpreting the results of the subgroup analyses, it needs to be considered that the sample size of the compared subgroup categories were unequal and different sample sizes may have influenced the *p* values.

Only three patients (4.3%) had vaginal pH at both D1 and D2. The mean change in D1 was − 2.1 (SD 1.2; range − 3 to − 1). Due to the very small sample analysed, the result is not meaningful.

Physicians rated the overall efficacy as ‘excellent/good’ for 96.7% of patients (*n* = 58), as ‘moderately effective’ for 1.7% (*n* = 1), and as ‘bad’ for 1.7% (*n* = 1) (FAS: *N*_valid_ = 60). The patients’ overall acceptance of local treatment with KadeFungin® Milchsäurekur was high. 94.1% (*n* = 64) of patients were ‘(very) satisfied’ and 5.9% (*n* = 4) ‘moderately satisfied’ (FAS: *N*_valid_ = 68). Similarly, handling was rated as ‘(very) easy’ by 94.2% (*n* = 65) of patients and as ‘moderate’ by 5.8% (*n* = 4) (FAS: *N*_valid_ = 69). The additional moisturizing effect was rated was ‘(very) pleasant’ by 72.5% (*n* = 50) of patients, as ‘neutral’ by 24.6% (*n* = 17), and as ‘unpleasant’ by 2.9% (*n* = 2) (FAS: *N*_valid_ = 69).

All AEs that occurred during the study were of mild intensity. No serious AEs were recorded. In total, 4 of the 72 patients in the SES (5.6%) experienced 6 AEs, thereof 1.4% with an ADE (mild application site pruritus) (Table 4). All six AEs had resolved by the end of the study.

**Table 4:** Overview of adverse events/adverse device effects reported during the observation period (SES: *N* = 72, 100%)

	Event(s) *n*	Patient(s) with at least one event *N* (%)
Total AEs/ADEs/patients with AEs/ADEs	**6**	**4 (5.6)**
AE (MedDRA) without causality to the medical device		
Infections and infestations	**2**	**2 (2.8)**
Cystitis (mild)	1	1 (1.4)
Subcutaneous abscess (mild)	1	1 (1.4)
Musculoskeletal and connective tissue disorders	**2**	**2 (2.8)**
Mobility decreased (mild)	1	1 (1.4)
Psychiatric disorders	**2**	**2 (2.8)**
Insomnia (mild)	1	1 (1.4)
Nervousness (mild)	1	1 (1.4)
AE with causality to the medical device (ADE		
General disorders and administration site condition		
Application site pain possible (mild)	**1** 1	**1 (1.4)** 1 (1.4)

The local tolerability of KadeFungin® Milchsäurekur was rated as ‘highly tolerable/tolerable’ by 98.6% (*n* = 68) of patients and as ‘moderately tolerable’ by 1.4% (*n* = 1) (FAS: *N*_valid_ = 69). Physicians rated the overall tolerability of the treatment as ‘excellent /good’ for 98.4% (*n* = 61) of patients and as ‘moderately tolerable’ for 1.6% (*n* = 1) (FAS: *N*_valid_ = 62).

## Discussion

To our knowledge, this is the first study to observe a preventive effect of a vaginal lactic acid gel in a larger group of women with a history of uncomplicated rUTIs during routine practice. The results suggest that vaginal lactic acid gel can reduce the rate of rUTIs when used for a short period every month. 66.7% of women had no further UTIs after a period of 4 months.

Uncomplicated rUTI in women is thought to arise from repeated ascending infection of the bladder by intestinal bacteria (usually *E. coli*) that colonise the vaginal vestibule and distal urethra in large numbers [10, 23, 24]. Vaginal culture studies have shown that women with rUTI also show a tendency to vaginal colonisation with *E. coli* during the asymptomatic periods [25]. Application of a vaginal lactic acid gel for short monthly courses with an appropriately amount of lactic acid could therefore not only favour beneficial *Lactobacillus*-dominated microbiota [26], but also limit the growth of *E. coli* by creating unfavourable growth conditions. Hudson et al. [27] investigated the relative effects of pH and lactic acid concentration on *E. coli* growth and were able to show a direct correlation.

There are different opinions about which is the most important antimicrobial factor produced by *Lactobacilli*: some groups describe that women with rUTI are deficient in H_2_O_2_-producing *Lactobacilli* [25, 28, 29], others describe lactic acid as the even more important antimicrobial factor [30]. Under the hypoxic conditions that prevail in the vagina, *Lactobacilli* would produce little or no H_2_O_2_.[30] Lactic acid was also shown to be a strong outer membrane disintegrating as well as permeabilising agent of gram-negative bacteria [31]. Swidsinski et al. [16] have demonstrated a positive effect of a vaginal lactic acid gel on acute *E. coli* cystitis. With a 4-week treatment period, not only the clinical symptoms disappeared in more than half of the patients, but also a bacteriostatic effect including morphological changes could be observed. Antibiotic treatment was not necessary. These results support our assumptions that vaginally applied lactic acid can limit *E. coli* growth and thus prevent a transfer of the uropathogen from the vagina to the urinary bladder. The research group did not find any preventive effect of lactic acid, but the study was focused on acute treatment and only a few patients used the vaginal gel for relapse prevention.

The effect of vaginal administration of lactic acid preparations to restore an optimal microbiota has mainly been shown for the treatment of bacterial vaginosis, in combination with an antibiotic or as single therapy [32–35]. Two recently published systematic review articles conclude that, despite methodological limitations and the need for large, rigorous randomised trials, vaginal lactic acid preparations are effective in the treatment of BV and can thus be considered as an alternative to antibiotics such as metronidazole [36, 37]. Comparable to our study, no major safety concerns were reported and the preparations were well tolerated [36]. While in our study, one woman experienced pain at the application site that was assessed as possibly related to the vaginal gel, women in the BV studies reported vaginal or vulvar irritation, itching, burning, redness and/or dryness [36].

Another non-antimicrobial, well-tolerated treatment option for the prevention of UTIs is the vaginal or oral use of probiotics mainly as *Lactobacillus* spp [15, 38–42]. Probiotics may prevent bladder invasion by inducing an immune response via the urethra or vagina and by colonising the vagina with beneficial bacteria. Furthermore, the antimicrobials might interfere with uropathogens [42]. Various effects of different strains on the growth or adhesion of uropathogens have been shown in in vitro studies [43, 44]. *Lactobacillus brevis* DT 24 was identified to produce a bacteriocin (Bacteriocin DT24), which effectively inhibits the growths of various pathogens [45]. The strain *Lactobacillus reuteri* CRL 1324 reduced the adhesion and internalisation of *E. coli* 275 into HeLa cells [46]. Under optimal conditions for lactic acid production, *Lactobacillus acidophilus* CRL 1259 inhibited the growth of *E. coli*.[47] Various strains have been investigated in randomised controlled or non-controlled trials over the past decades for their efficacy in preventing urinary tract infections. Despite the promising in vitro results [39], the clinical trial data are inconclusive [15, 38]. While some studies have shown no effect on the recurrence rate of UTIs [48–50], others have shown a reduction in the number of potential pathogenic bacteria and yeasts colonising the vagina in healthy women [51] or a reduction in the recurrence of UTI when administering *Lactobacillus rhamnosus* GR-1 and *Lactobacillus fermentum* RC-14 in women with a history of UTI [52]. Beerepoot et al. [53] reported a reduction in the mean number of UTIs in the previous year from 6.8 to 3.3 in postmenopausal women taking *Lactobacillus rhamnosus* GR-1 and *Lactobacillus fermentum* RC-14 twice daily for 12 months, whereby the predefined non-inferiority threshold was not reached compared to long-term treatment with trimethoprim-sulfamethoxazole. However, unlike trimethoprim-sulfamethoxazole, *Lactobacilli* do not increase antibiotic resistance.

Our study lacks a control group. Stapleton [28] reported that in 100 premenopausal women who initially received *Lactobacillus crispatus* CTV-05 or placebo daily for 5 days, then once weekly for 10 weeks, recurrent UTIs occurred in 15% of women receiving the probiotic compared with 27% of women receiving placebo. The absolute numbers of UTI outcome in the probiotic group were similar to our results at month two in the CC group, where 16.7% had a recurrent UTI. This supports our findings. They could also show that women who received probiotic achieved a high-level *Lactobacillus crispatus* vaginal colonization pattern, which was not shown in other studies with different strains.

However, the treatment with lactic acid gel bypasses the indeterminate search for a suitable probiotic strain and risk of insufficient colonisation. Ideally, the negative feedback loop during rUTIs, in which pathogens such as *E. coli* increase pH-levels, is interrupted and reversed by the treatment with lactic acid.

The presented observational study offers valuable insights from real-world treatment of a highly prevalent urogynecological disorder. Study results suggest that the treatment with vaginal lactic acid gel could prevent and delay the occurrence of urinary tract infections. The assessment of the results was consistent between patients and treating physicians. This further substantiates the results of a beneficial effect of preventing and delaying rUTIs with lactic acid gel treatment.

In uncomplicated cases of recurrent UTIs, the intermittent use of a vaginal lactic acid gel for relapse prevention could therefore reduce the excessive long-term use of antibiotics.

The study is limited in some regards. Treatment/observational time was limited to 4 months to achieve the highest possible compliance of the patients in filling out the diaries. Nevertheless, with a mean of four UTIs in the last 12 months, an observation period of 4 months is an adequate period to evaluate improvements. However, an extension of the period to 6 or 12 months would be preferable. Further trials and studies are still warranted to improve our understanding of ideal treatment options.

## Conclusion

Intermittent short courses of intravaginal treatment with lactic acid gel (KadeFungin® Milchsäurekur), applied 2–3 days after each menstruation, may be beneficial for the resilience against uropathogens and reduce the rate of reinfection in UTI-prone women. Intravaginal treatment with the lactic acid gel was overall very well tolerated by patients and was generally assessed very positively by the patients. The physicians’ assessments corroborated these findings.

## Data Availability

All relevant data are within the manuscript.
