# Correction to: Vaginal treatment with lactic acid gel delays relapses in recurrent urinary tract infections: results from an open, multicentre observational study

**DOI:** 10.1007/s00404-021-06093-9

**Published:** 2021-05-15

**Authors:** Ruth Diebold, Bettina Schopf, Holger Stammer, Werner Mendling

**Affiliations:** 1 Dr. Kade Pharmazeutische Fabrik GmbH, Berlin, Germany; 2 Pharmalog Institut für klinische Forschung GmbH, Ismaning, Germany; 3 German Centre for Infections in Obstetrics and Gynecology, Wuppertal, Germany

## Correction to: Archives of Gynecology and Obstetrics 10.1007/s00404-021-06040-8

In the original article published the conflicts of interest were not used in the same wording as in the manuscript submitted. The correct statement is given below:

Author R Diebold is an employee of DR. KADE Pharmazeutische Fabrik GmbH, Berlin, Germany and received support in form of salary. The authors B Schopf and H Stammer are employees of the Clinical Research Organistation Pharmalog Institut für klinische Forschung GmbH, which was contracted by DR. KADE. The scientific study coordinator W Mendling received honorarium for this work as well as speaker honoraria at scientific training events organized by the funder.

